# Airway reversibility in asthma and phenotypes of Th2-biomarkers, lung function and disease control

**DOI:** 10.1186/s13223-018-0315-0

**Published:** 2018-12-27

**Authors:** Jianghong Wei, Libing Ma, Jiying Wang, Qing Xu, Meixi Chen, Ming Jiang, Miao Luo, Jingjie Wu, Weiwei She, Shuyuan Chu, Biwen Mo

**Affiliations:** 1 Department of Respiratory and Critical Care Medicine, Affiliated Hospital of Guilin Medical University, Guilin, 541001 Guangxi China; 2 Laboratory of Respiratory Diseases, Affiliated Hospital of Guilin Medical University, Guilin, 541001 Guangxi China

**Keywords:** Asthma, Airway reversibility, Th2-biomarkers, Lung function, Asthma control

## Abstract

**Background:**

High bronchodilator reversibility in adult asthma is associated with distinct clinical characteristics. In this study, we aim to make a comparison with T-helper 2 (Th2)-related biomarkers, lung function and asthma control between asthmatic patients with high airway reversibility (HR) and low airway reversibility (LR).

**Methods:**

Patients with asthma diagnosed by pulmonologist according to Global Initiative for Asthma guidelines were recruited from the outpatient department of our hospital from August 2014 to July 2017. Patients were divided into HR and LR subgroups based on their response to bronchodilators of lung function (HR = Δforced expiratory volume in one second (FEV1) postbronchodilator ≥ 20%). Blood eosinophil count and serum IgE level, which are biomarkers of T-helper (Th)-2 phenotypes, were detected for patients. Asthma Control Test (ACT) was used to assess asthma control after the first-month initial treatment.

**Results:**

A total of 265 patients with asthma were followed 1 month after initial treatment. HR group shows a higher level of Th2-high biomarkers (blood eosinophil count (10^9/L): 0.49 ± 0.28 vs 0.36 ± 0.19, P < 0.01; IgE (ng/ml): 1306 ± 842 vs 413 ± 261, P < 0.01), lower baseline lung function (FEV1%pred: 51.91 ± 19.34% vs 60.42 ± 19.22%, P < 0.01; forced expiratory flow (FEF)25–75: 0.76 ± 0.37 vs 1.00 ± 0.67, P < 0.01; FEF25–75%pred: 21.15 ± 10.09% vs 29.06 ± 16.50%, P < 0.01), and better asthma control (ACT score: 22 ± 4 vs 20 ± 4, P = 0.01) than LR group. HR was associated with a decreased risk of uncontrolled asthma after the first-month initial treatment (adjusted OR: 0.12 [95% confidence intervals: 0.03–0.50]).

**Conclusions:**

HR is a physiologic indicator of lower lung function and severer small airway obstruction, and is more related with an increased level of Th2-biomarkers than LR. Moreover, HR may indicate controlled asthma after the first-month initial treatment. This finding may contribute to identification of asthma endotype.

## Background

Airway bronchodilator reversibility is the characteristic that differentiates asthma population from patients with irreversible obstructive lung diseases [1]. It has been emerged as a characteristic to categorize asthma patients into different phenotype [2], and a physiologic biomarker associated with co-morbidities of asthma patients [3]. Interestingly, the high airway bronchodilator reversibility was found as a physiologic indicator for reduced lung function, and was associated with elevated Th2-biomarkers [4]. Thus, airway bronchodilator reversibility may be a crosslinking point in understanding the diversity of asthma endotype, and then identify profiles to guide treatment [5]. However, that previous study didn’t investigate obstruction in small airway, or asthma control after the initial treatment according to the Global Initiative for Asthma guidelines (GINA) alone [1]. Therefore, we conducted this hospital-based cohort study to investigate immune pathway biomarkers, obstruction in small airway, and disease control after the initial treatment in asthma patients with high or low airway reversibility.

## Methods

From August 2014 to July 2017, adult patients with asthma diagnosed by pulmonologists at the first time according to the definition of GINA [1] were recruited in the study from the Affiliated Hospital of Guilin Medical University, Guilin, China. Blood eosinophil count and serum IgE level were tested. Asthma control was assessed in terms of Asthma Control Test (ACT) after the first-month initial treatment with a face-to-face interview by pulmonologists [6, 7]. The study protocol was approved by the Institutional Review Board at the Affiliated Hospital of Guilin Medical University, and conformed to the declaration of Helsinki. Written informed consent was obtained from each subject.

Inclusion criteria in the present study were as following: (1) age between 18 and 65 years, (2) forced expiratory volume in one second (FEV1) % predicted less than 80%, (3) reversibility in FEV1 12% (and at least 200 ml) following administration of a short-acting β-agonist, (4) no evidence of active infection, (5) no medical conditions associated with immune suppression. Patients were excluded if they had chronic obstructive pulmonary disease (COPD) or asthma-COPD overlap [8], had a history of intubation within 3 years of enrollment, or had obstructive sleep apnea.

Subjects were classified into high airway reversibility (HR) group and low airway reversibility (LR) group. The HR group included patients with a ≥ 20% increase in FEV1 following administration of a short-acting bronchodilator during screening and baseline pulmonary function testing. The LR group included those with reversibility below that level [2].

Group data were expressed as the mean ± standard deviation (SD). Significant differences were evaluated using independent-samples *t* test or Chi square test. The associations between asthma control and clinical characteristics were explored with unconditional logistic regression models with LOGISTIC procedure in SAS 9.4 (SAS Institute Inc., Cary, North Carolina, USA). The results were presented as odds ratios (OR) and 95% confidence intervals (CI). P values < 0.05 were considered to be statistically significant.

## Results

**Fig. 1:**
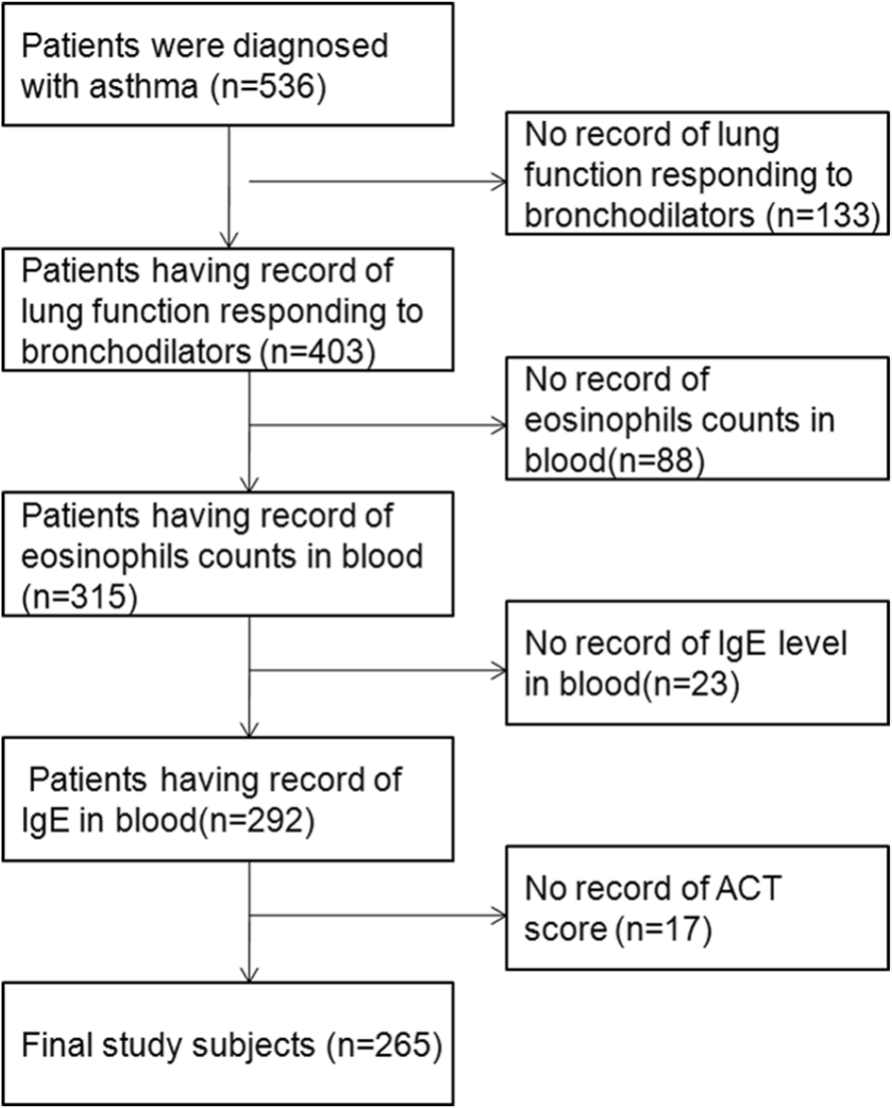
Selection of the study population

Figure 1 shows the subject selection process. We excluded subjects if they had no record of lung function responding to bronchodilators, blood eosinophil count, serum IgE level, or ACT score. A total of 265 subjects were selected for final analyses.

Demographics characteristics for all patients were summarized in Table 1. HR and LR groups were similar in percentages of male, smoker and subjects having history of allergy, age and body-mass index (BMI). All patients were received inhaled corticosteroid (ICS) combined long-acting inhaled β2-agonist (LABA) as initial treatment.

**Table 1:** Comparison of patients between HR and LR groups

Variable	HR group (n = 60)	LR group (n = 205)	P value
Gender (male)	30 (50.0%)	82 (40.0%)	0.17
Age (years)	45 ± 10	47 ± 13	0.23
BMI (kg/m2)	22.80 ± 3.34	23.50 ± 4.07	0.23
Smoker (yes)	21 (35.0%)	90 (43.9%)	0.22
Self-reported history of allergy (yes)	7 (11.7%)	42 (20.5%)	0.12
Eosinophil count in blood (10^9/L)	0.49 ± 0.28	0.36 ± 0.19	< 0.01
IgE in blood (ng/ml)	1306.0 ± 841.5	413.4 ± 261.6	< 0.01
High-dose ICS+LABA	2 (3.3%)	3 (1.5%)	0.69
Lung function test			
Pre inhaling short-acting bronchodilator			
FVC (L)	3.01 ± 2.62	2.69 ± 0.84	0.14
FEV1 (L)	1.52 ± 0.62	1.69 ± 0.65	0.08
FEV1%pred (%)	51.91 ± 19.34	60.42 ± 19.22	< 0.01
FEV1/FVC (%)	55.43 ± 11.97	61.94 ± 12.59	< 0.01
PEF (L/s)	3.60 ± 1.49	3.88 ± 1.61	0.23
PEF%pred	43.70 ± 16.80	50.59 ± 18.86	0.01
FEF50 (L/s)	0.97 ± 0.50	1.23 ± 0.75	< 0.01
FEF50%pred (%)	23.05 ± 12.18	30.75 ± 17.26	< 0.01
FEF75 (L/s)	0.39 ± 0.40	0.69 ± 1.07	< 0.01
FEF75%pred (%)	19.05 ± 9.16	25.96 ± 16.08	< 0.01
FEF25–75	0.76 ± 0.37	1.00 ± 0.67	< 0.01
FEF25–75%pred (%)	21.15 ± 10.09	29.06 ± 16.50	< 0.01
Post inhaling short-acting bronchodilator			
FVC (L)	2.73 ± 0.99	2.79 ± 0.85	0.67
FEV1 (L)	2.11 ± 0.98	1.84 ± 0.68	0.02
FEV1%pred (%)	72.45 ± 32.46	67.56 ± 26.88	0.23
FEV1/FVC (%)	81.69 ± 38.48	65.73 ± 16.38	< 0.01
PEF (L/s)	4.12 ± 1.54	4.12 ± 1.69	0.98
PEF%pred	50.09 ± 17.85	53.12 ± 18.96	0.27
FEF50 (L/s)	1.38 ± 0.75	1.46 ± 0.81	0.51
FEF50%pred (%)	33.12 ± 16.20	36.69 ± 18.64	0.15
FEF75 (L/s)	0.58 ± 0.59	0.76 ± 0.97	0.08
FEF75%pred (%)	27.84 ± 14.14	31.28 ± 18.86	0.13
FEF25-75	1.15 ± 0.65	1.50 ± 4.40	0.28
FEF25–75%pred (%)	30.99 ± 16.96	44.60 ± 136.36	0.17
FEV1 change^a^	30.13 ± 9.69	8.83 ± 9.11	< 0.01
ACT score	22 ± 4	20 ± 4	0.01

*HR* high airway reversibility, *LR* low airway reversibility, *BMI* body mass index, *ICS* inhaled corticosteroid, *LABA* long-acting b-agonist, *FEV1* forced expiratory volume in 1 s, *FVC* forced vital capacity, *PEF* peak expiratory flow, *FEF* forced expiratory flow, *%pred* % predicted, *ACT* asthma control test

^a^ FEV1 change between pre- and post-inhaling short-acting bronchodilator

Moreover, Table 1 illustrates the difference of Th-2 phenotypes, lung function and asthma control between HR and LR groups. In HR group, eosinophil count in blood and IgE level in serum were higher than those in LR group (eosinophil count (10^9/L): 0.49 ± 0.28 vs 0.36 ± 0.19; IgE (ng/ml): 1306.0 ± 841.5 vs 413.4 ± 261.6. All P values < 0.01). Moreover, the base lung function in HR group was worse than that in LR group (FEV1%pred: 51.91 ± 19.34% vs 60.42 ± 19.22%; forced expiratory flow (FEF)25–75: 0.76 ± 0.37 vs 1.00 ± 0.67; FEF25–75%pred: 21.15 ± 10.09% vs 29.06 ± 16.50%. All P values < 0.01). In addition, HR group was showed better asthma control than LR group after the first-month initial treatment (ACT score: 22 ± 4 vs 20 ± 4, P = 0.01).

We further explored the association between HR and asthma control after the first-month initial treatment. Table 2 shows that HR was associated with a decreased risk of uncontrolled asthma after the first-month initial treatment (adjusted OR for ACT score < 20: 0.12 [95% CI 0.03–0.50]). We furthermore assessed the association between asthma control and obstruction in small airway after inhaling short-acting bronchodilator. We found that FEF25–75 was associated with a decreased risk of uncontrolled or partly controlled asthma (adjusted OR for ACT score < 20: 0.07 [95% CI 0.01–0.73]; adjusted OR for ACT score 20–24: 0.03 [95% CI 0.01–0.71]).

**Table 2:** Adjusted and unadjusted relative risks of asthma control after the first-month initial treatment

Exposure categories	Unadjusted OR	95% CI	P values	Adjusted OR	95% CI	P values
Asthma uncontrolled (n = 105)						
HR^a^	0.36	0.16–0.82	0.02	0.12	0.03–0.50	< 0.01
Eosinophil count in blood (10^9/L)^a^	1.10	0.18–6.58	0.92	2.36	0.23–23.96	0.47
IgE in blood (ng/ml)^a^	1.00	0.99–1.00	0.29	1.00	0.99–1.00	0.89
FEF75 (L/s)^b, c^	1.31	0.77–2.24	0.32	0.82	0.41–1.63	0.57
FEF75%pred (%)^b, c^	1.01	0.99–1.04	0.24	1.01	0.96–1.06	0.81
FEF25–75^b, c^	1.57	0.91–2.73	0.11	0.07	0.01–0.73	0.03
FEF25–75%pred (%)^b, c^	1.02	1.02–1.05	0.04	1.09	1.01–1.18	0.02
Asthma partly controlled (n = 120)						
HR^a^	0.59	0.27–1.28	0.18	0.35	0.10–1.22	0.10
Eosinophil count (10^9/L)^a^	1.79	0.32–9.97	0.51	2.85	0.31–26.12	0.36
IgE (ng/ml)^a^	1.00	0.99–1.00	0.93	1.00	0.99–1.001	0.93
FEF75 (L/s)^b, c^	1.30	0.76–2.20	0.34	0.84	0.42–1.65	0.60
FEF75%pred (%)^b, c^	1.02	0.99–1.04	0.18	1.01	0.95–1.06	0.82
FEF25–75^b, c^	1.52	0.87–2.62	0.14	0.03	0.01–0.71	0.03
FEF25–75%pred (%)^b, c^	1.02	1.00–1.04	0.06	1.07	1.00–1.15	0.06

*HR* high airway reversibility, *ACT* asthma control test, *FEF* forced expiratory flow, *%pred* % predicted, *FEV1* forced expiratory volume in 1 s, *FVC* forced vital capacity

Asthma uncontrolled: ACT < 20, Asthma partly controlled: 20 ≤ ACT ≤ 24, Asthma controlled: ACT = 25

^a^ Adjusted for age, BMI, eosinophil count, IgE, HR, FEV1, FEV1%pred, FVC, FEV1/FVC, FEV1-post inhaling short-acting bronchodilator, FVC-post inhaling short-acting bronchodilator, FEV1/FVC-post inhaling short-acting bronchodilator

^b^ Adjusted for age, BMI, eosinophil count, IgE, HR, FEF50, FEF50%pred, FEF75, FEF75%pred, FEF25–75, FEF25–75%pred

^c^ Lung function after inhaling short-acting bronchodilator

## Discussions

In our study, HR was more frequently associated with a higher level of Th2-biomarkers, lower lung function in baseline, and better asthma control after the first-month initial treatment than LR. Furthermore, HR and high FEF25–75 after inhaling short-acting bronchodilator were respectively associated with better asthma control. It may contribute to identifying the endotype of asthma by further clarifying the relationship between airway bronchodilator reversibility, obstruction in small airway, Th2-biomarkers and disease control after initial treatment.

In the present study, asthmatic patients with HR were showed a higher level of Th2-biomarkers than LR group. That finding was similar with previous report [4]. Moreover, in comparison with that previous study, we found that not only serum IgE level but also blood eosinophil count of patients with HR was higher than those with LR. That may be mainly due to different characteristics of our subjects from that previous study. In our study, all patients received ICS combined LABA treatment. ICS could affect circulating eosinophil counts and cytokine expression [9]. In addition, Chinese demographics and clinical characteristics of populations in our study may partly contribute to our different findings. In our study, Chinese patients were showed a lower baseline lung function, a higher level of Th2-biomarkers than Europeans in previous study [4]. Thus, Th2-biomarkers may be more related with HR endotype than LR in Chinese patients with asthma.

Interestingly, it didn’t find an association between Th2 biomarker and asthma control after the first-month initial treatment in our study. Previous study found that asthmatic patients with higher blood eosinophil counts fared poorer asthma control [10]. However, that study followed subjects for a long term, and didn’t combine with the phenotype of HR. Thus, our study suggested that for patients with HR, higher Th2-biomarkers may not indicate poorer disease control after a short-term initial treatment. Blood eosinophil counts as a biomarker of asthma control in patients with HR phenotype may be combined with other biomarkers. Future study with larger sample size is needed to confirm our findings.

FEF50, FEF75 and FEF25–75 in baseline of patients with HR were less than those with LR in our study. FEF75 and FEF25–75 could accurately reflect flow at low lung volumes, which is helpful to showing small airway obstruction in early stage of obstructive lung disease [11, 12]. Particularly, FEF25–75 is a sensitive indicator for obstructive small airway disease [13]. Thus, asthmatic patients with HR may have a higher risk for small airway obstruction than those with LR. Furthermore, we assessed the association between asthma control after initial treatment and FEF75 or FEF25–75 post-inhaling bronchodilator. We found that FEF25–75 post-inhaling bronchodilator was associated with a decreased risk of poor asthma control, suggesting that high FEF25–75 post-inhaling bronchodilator may indicate better asthma control. Since FEF25–75 is an effective indicator for asthma control [14], FEF25–75 post-inhaling bronchodilator may be a desirable indicator for asthma control after short-term initial treatment in the endotype of HR.

Furthermore, the present study showed that HR was associated with a decreased risk of poor asthma control after the first-month initial treatment. In contrast, previous study found that patients with higher airway reversibility were showed worse controll of asthma at 12 month-follow-up [15]. These findings suggested that asthmatic patients with HR may have a better response than those with LR at the beginning of initial treatment with ICS combined with LABA. However, the long-term response to that treatment may be not good in patients with HR as previous reported [15]. The factors are not well clarified. It may be partly related with complex pathogenesis and progress of asthma, variously adjustment in therapeutic strategies during long-term treatment, or patients’ incompliance with treatment. Thus, for patients with HR, it may be particularly important to regularly monitor disease control and then adjust therapeutic strategy in a long term.

We acknowledge that our study has limitations. The patients were followed only 1 month after initial treatment. Although we can’t explore asthma control of HR group in a long-term, previous study reported different findings on asthma control in patients with HR for a long-term from a short-term [15]. Those indicated that there may be dynamic changes of asthma control during a long period of treatment. Moreover, atopy test was absent in our study. Thus, we couldn’t explore the relationship between atopy and HR endotype. Even though, the self-reported history of allergy was not significant different between HR and LR groups when demographic characteristics were well matched.

## Conclusions

In conclusion, HR is a physiologic indicator of lower lung function, particularly small airway obstruction, and is more related with an increased level of Th2-biomarkers than LR. Moreover, HR indicates well asthma control after the first-month initial treatment. Those findings may be help to identify the endotype of asthma.

## Abbreviations

GINA: Global Initiative for Asthma guidelines
ACT: Asthma Control Test
FEV1: forced expiratory volume in one second
Th2: T-helper 2
COPD: chronic obstructive pulmonary disease
HR: high airway reversibility
LR: low airway reversibility
OR: odds ratios
CI: confidence intervals
SD: standard deviation
BMI: body-mass index
ICS: inhaled corticosteroid
LABA: long-acting inhaled β2-agonist
FEF: forced expiratory flow
